# Analysis of the obstetrician's posture and movements during a simulated forceps delivery

**DOI:** 10.1186/s12884-024-06457-4

**Published:** 2024-04-08

**Authors:** Manon Sorel, Bertrand Gachon, Perrine Coste-Mazeau, Yves Aubard, Fabrice Pierre, Laetitia Fradet

**Affiliations:** 1 Department of Obstetrics and Gynecology, University of Poitiers, University Hospital Center of Poitiers, Poitiers, 86000 France; 2 Pprime Institute UPR 3346-CNRS, University of Poitiers, Axe RoBioSS, Poitiers, 86073 France; 3 Clinical Investigation Center, INSERM CIC 1402, Poitiers, 86000 France; 4 Department of Obstetrics and Gynecology, University of Limoges, University Hospital Center of Limoges, Limoges, 87000 France

**Keywords:** Forceps delivery, Motion capture, Obstetrical biomechanics, Traction forces, Training simulation

## Abstract

**Background:**

The objective of this study was to identify and qualify, by means of a three-dimensional kinematic analysis, the postures and movements of obstetricians during a simulated forceps birth, and then to study the association of the obstetricians’ experience with the technique adopted.

**Method:**

Fifty-seven volunteer obstetricians, 20 from the Limoges and 37 from the Poitiers University hospitals, were included in this multi-centric study. They were classified into 3 groups: beginners, intermediates, and experts, beginners having performed fewer than 10 forceps deliveries in real conditions, intermediates between 10 and 100, and experts more than 100. The posture and movements of the obstetricians were recorded between December 2020 and March 2021 using an optoelectronic motion capture system during simulated forceps births. Joint angles qualifying these postures and movements were analysed between the three phases of the foetal traction. These phases were defined by the passage of a virtual point associated with the forceps blade through two anatomical planes: the mid-pelvis and the pelvic outlet. Then, a consolidated ascending hierarchical classification (AHC) was applied to these data in order to objectify the existence of groups of similar behaviours.

**Results:**

The AHC distinguished four different postures adopted when crossing the first plane and three different traction techniques. 48% of the beginners adopted one of the two raised posture, 22% being raised without trunk flexion and 26% raised with trunk flexion. Conversely, 58% of the experts positioned themselves in a “chevalier servant” posture (going down on one knee) and 25% in a “squatting” posture before initiating traction. The results also show that the joint movement amplitude tends to reduce with the level of expertise.

**Conclusion:**

Forceps delivery was performed in different ways, with the experienced obstetricians favouring postures that enabled observation at the level of the maternal perineum and techniques reducing movement amplitude. The first perspective of this work is to relate these different techniques to the traction force generated. The results of these studies have the potential to contribute to the training of obstetricians in forceps delivery, and to improve the safety of women and newborns.

**Supplementary Information:**

The online version contains supplementary material available at 10.1186/s12884-024-06457-4.

## Introduction

Operative vaginal deliveries (by forceps, spatulas, or vacuum extractors) are currently on the decline in delivery rooms [1]. The rate of operative vaginal deliveries varies widely between countries, from 0.5% in Romania to 16.4% in Ireland, while the median rate in Europe is 7.5% [2] and 5% in the United States [1]. These deliveries are also the main risk factor for obstetric perineal trauma, in particular, obstetric injury to the anal sphincter and disinsertion of the levator ani muscle [3], the risk being increased when the instruments are forceps [4, 5]. Currently, the share of instrumental deliveries by forceps is decreasing in favour of vacuum delivery. The 2021 national perinatal survey reports indeed 20.9% forceps and 60.2% vacuum deliveries compared to 27.6% and 49.8% respectively in 2016 [6]. Accompanying this reduction of instrumental deliveries by forceps is a loss of practice and training [7, 8]. In spite of this, forceps are the only instrument that enables to perform traction, which might be necessary, for example, in the event of the absence or insufficiency of maternal pushing.

Moreover, it has been clearly established that a less-trained doctor risks performing the technique in an improper manner, which consequently risks inducing serious and avoidable maternal injuries [9]. The significant drop in the use of forceps inevitably leads to a drop in the transmission of expertise by association and, consequently, to a progressive decrease in the number of expert obstetricians. In order to increase maternal and foetal safety, there is thus a need to maintain the use of forceps by promoting learning and training, which can be performed on mannequins. However, only a few pieces of information are provided in the literature about what is essential to ensure a safe and successful forceps delivery.

Several authors have shown that the intensity of the forces produced and the number of tractions applied to the foetal head are associated with the occurrence of maternal-foetal lesions [10–12]. A symmetrical application of the blades on the foetal skull bones also increases the surface area of the application of the force, which reduces the stress applied to the foetal bones [13]. If these results underline the importance of the forces applied to the foetus, little is known about how the obstetrician should proceed to achieve adequate force production.

With the aim of favouring the control of the traction force during forceps delivery, one study proposed to train obstetricians on a simulator [14]. The results showed that training on a simulator with visual feedback helped junior obstetricians to no longer exceed a safety threshold set at 200N and also that the intensity of the traction force was greater when sitting than when standing [14]. However, in this study, no recommendations have been made on how to proceed from the point of view of movement or from the point of the posture to adopt to produce and control the traction force.

To our knowledge, only two studies provide some information about posture and movements. In Matsumoto et al.’ study [12], one can read that “To avoid falling, he (the obstetrician) adopted a fighter’s stance with a wide stance and slightly bent knees.” whereas in Evanson and Riggs’ study [15] is written that “Forceps are applied from below the foetal head while sitting. Traction force originates from the forearms, not the chest.». For the first study, it implies that the motivation for the posture is not the traction force production but rather the obstetrician’s balance. For the second, if it is not clear whether the obstetrician should remain seated after forceps placement, this is the only paper that mentions how the traction movement should be performed. One can also note that the two postures evoked are substantially different.

Studies are therefore essential to better understand the mechanisms of forceps delivery and to identify the best technique in terms of the obstetrician's posture and movement when performing this intervention, so that the safest technique for both mother and child can be taught. The motivation to set up this project, the ultimate aim of which was to define whether there is an optimal technique when performing a forceps delivery, arose from the following factors: 1) the absence of recommendations on which technique to adopt when performing a delivery by forceps, from the point of view of the obstetrician’s posture and movements; 2) the potential occurrence of serious maternal injuries induced by these deliveries; and 3) the interest in maintaining the use of forceps.

In this first study of the project, the objective was to identify and describe, by means of a three-dimensional kinematic analysis, the posture and movements of obstetricians during a forceps delivery simulation, and to measure the association of the level of experience with the techniques found.

## Material and method

### Study population and inclusion criteria

An experimental bi-centric study was conducted between December 2020 and March 2021.

Email-based recruitment included an e-mail and a flyer describing the protocol and inviting participation. This email was sent to all the obstetricians from the two centres. These two centres were chosen firstly because the practice of instrumental delivery by forceps is performed there, which ensures that all the obstetricians had done at least once a forceps delivery, and, secondly, because the principal type of instrumental delivery is not the same, which should provide a diversity in experience. The instrument most commonly used in Limoges is indeed the forceps, whereas in Poitiers it is the vacuum extractor.

We classified obstetricians into three groups: beginners, intermediate, and experienced. Beginners had performed fewer than 10 forceps deliveries in real conditions (excluding simulation), intermediates between 10 and 100, and experts more than 100.

As no previous study has proposed analysing the movements of obstetricians during forceps delivery, the power analysis was based on the intensity of the traction forces measured by Leslie et al. [14]. More specifically, the mean force and the standard deviation produced by the women (45.5 lb ± 10.9) while standing and the safety range of 35 lb were taken, these values being the most conservative in the paper [14]. This resulted in 10 subjects per group for an alpha of 0.05 and a power of 80%. Finally, fifty-seven doctors were included on a voluntary basis: 20 from the Limoges (65%) and 37 from the Poitiers University hospitals (35%).

### Material

The forceps delivery was simulated on a mannequin composed of a maternal pelvis and a foetus (MODEL-med® Sophie and Sophie’s Mum Birth Simulator Version 4.0, Melbourne, Australia). The forceps, used in both centres, were Suzor type with parallel branches.

**Fig. 1:**
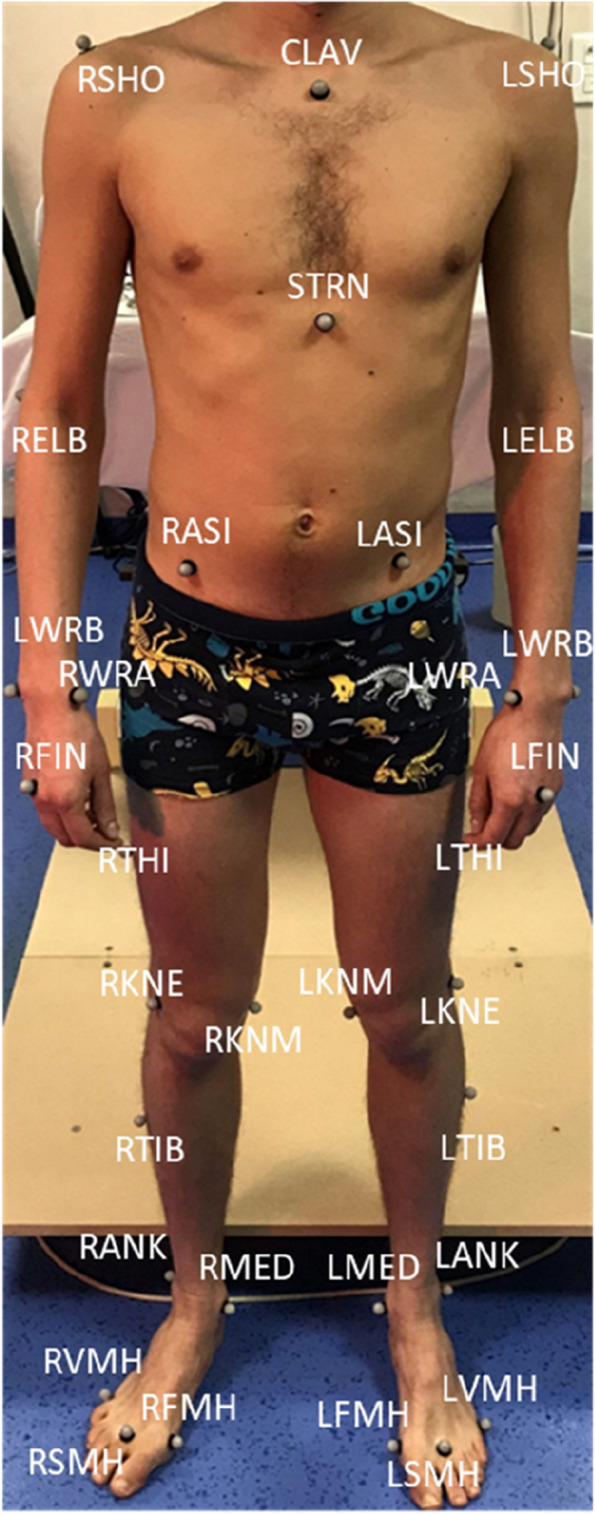
Marker set-up following the Conventional Gait Model 2.4. Four markers are positioned on the head, two on the back, and two on the pelvis [16]

To determine the obstetricians' postures and movements, a delivery room was equipped with 10 infrared cameras clocked at 100 Hz (Vicon, Oxford Metrics, UK) and arranged around the delivery table. The role of this system was to capture the trajectories of reflective markers placed on the obstetricians. These 42 reflective markers were positioned on anatomical landmarks according to the Conventional Gait model, full body version 2.4 [16], as depicted in Fig. 1. With this model, the human body can be divided into 14 segments (feet, lower and upper legs, trunk, forearms, upper-arm, hand, and head) and the angles between two consecutive segments can be computed [16].

**Fig. 2:**
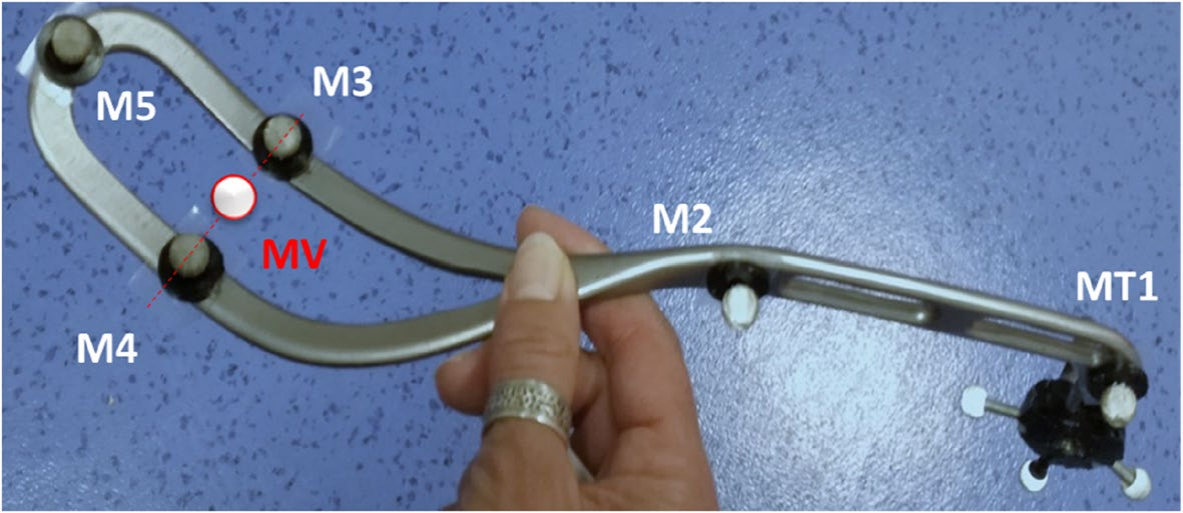
Suzor forceps instrumented with markers placed at the level of the handle extremity (M1), the heel (M2), the inferior (M3) and superior part of the blade (M4), and the toe of each blade (M5). A cluster of markers (MT1), used to define the technical coordinate system, was also placed at the handle extremity. The indicator in red represents the virtual marker (MV), in the middle of markers 3 and 4, used to define the different delivery phases

**Fig. 3:**
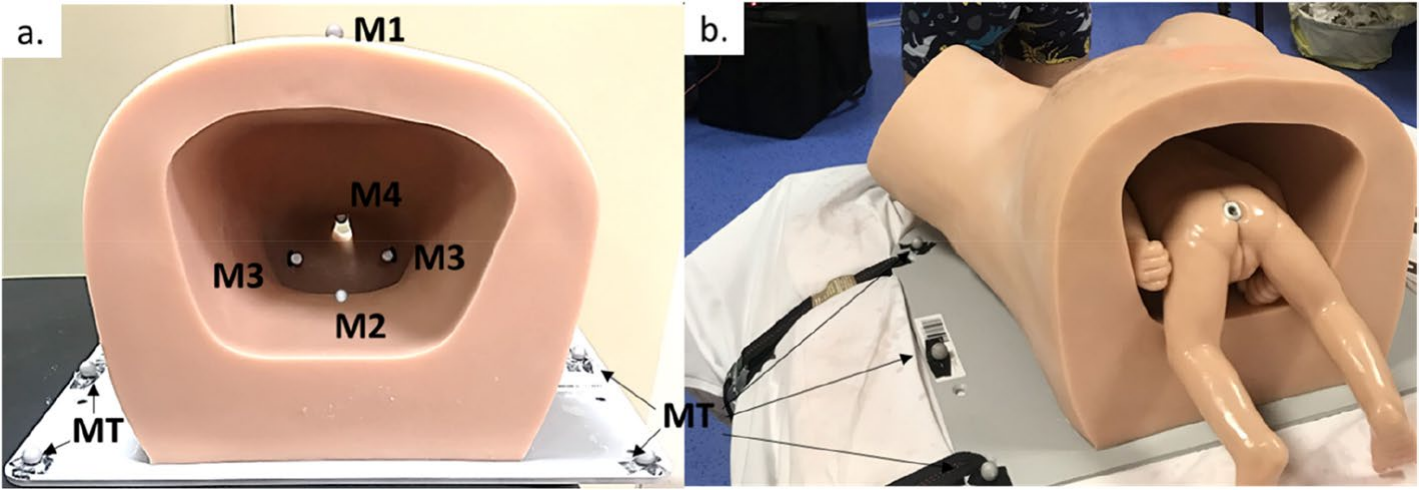
Mannequin instrumented with technical markers. **a** shows a mannequin instrumented with markers placed on the umbilicus (M1), the distal end of the sacrum (M2), the two sciatic spines (M3), and the lower edge of the pubic symphysis (M4). **a** and **b** show markers (MT) positioned on the mannequin's board to define a technical coordinate system necessary to reconstruct the maternal planes of interest

Three markers were also placed on the forceps (Fig. 2) and six others on the base of the maternal mannequin (Fig. 3) in order to constitute so-called “technical markers” as explained in detail in our previous article describing the method used [17]. These technical markers were used to define three coordinate systems: two associated with each blade of the forceps, and one associated with the maternal pelvis, which made it possible to reconstruct the position of necessary landmarks where these landmarks could not be seen by the cameras during the intervention. The markers located on the blades or pelvis anatomical points, such as the sacrum, could not be tracked by the cameras during the intervention because they were inside the mannequin.

### Protocol

Before each manipulation, the maternal pelvis was securely attached using two straps to the edge of a height-adjustable delivery table. Next, the obstetrician’s anthropometric data that was necessary for the model, such as weight and height and the dimensions of segments including the width of the knees, ankles, elbow, and wrist, were recorded [18, 19]. The reflective markers were then placed on the obstetrician.

The foetal mannequin was manually oriented in a cephalic presentation in an antero-posterior diameter, with the occiput in sight of the pubic symphysis. The foetal head was placed above the + 2 cm station according to the ACOG classification. [20]. This position was chosen because it is the most frequent.

Then, the obstetricians were able to adjust the height of the delivery table according to their personal clinical habits. Time was allowed before the recordings so that each obstetrician could become familiar with the equipment. The obstetricians then carried out the forceps delivery according to their usual practice: placing the two forceps blades on the foetal head, pulling the foetus, removing the instrument, and finalizing the simulated delivery.

Each obstetrician performed three simulations of forceps delivery in order to prevent intra-individual variability and anticipate eventual errors in data collection due to hidden markers.

### Data treatments

The Conventional Gait Model version 2.4 [16] was applied to identify the joint centres and the rotation axes necessary for the definition of the body-segment coordinate systems required to calculate the rotations between adjacent segments. These rotations were defined using Euler angles according to the sequence flexion–extension, abduction–adduction, and internal–external rotation, following the recommendations formulated by the International Society of Biomechanics [18, 19].

**Fig. 4:**
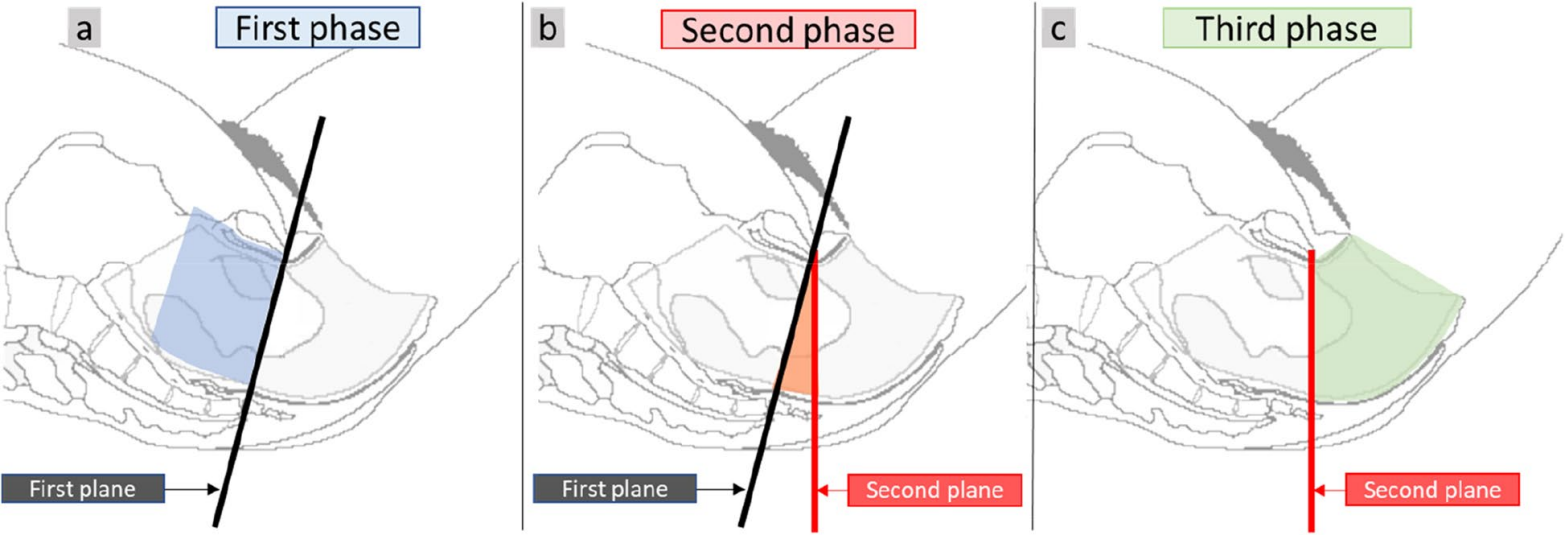
Definition of the plans and the three pulling phases. **a** The first plane was defined by the lower border of the pubic symphysis and the coccyx. **b** The second plane was defined by the lower border of the pubic symphysis and the two sciatic spines. The crossing of these planes by the virtual point, presumed to coincide with the foetal cephalic presentation, defines the first phase (**a**: from the beginning of the traction until the passage of the first plane), the second phase (**b**: from the first plane until the passage of the second plane), and the third phase (**c**: from the second plane until traction stops)

As mentioned above, direct visualization of the trajectory of the foetal head in the maternal pelvis was impossible since it was not visible to the cameras. Consequently, the hypothesis was made that the foetal head coincided with the centre of two virtual points located in the middle of the inferior and superior parts of each forceps blade (markers 3 and 4 in Fig. 2). This virtual point was used to estimate the crossing of anatomical planes by the foetal head. The first plane, corresponding to the mid-pelvis, was defined as the plane passing through the sciatic spines and the distal end of the sacrum. The second plane, equivalent to the pelvis outlet, was defined as the plane orthogonal to the plane of the support passing through the lower edge of the pubic symphysis (Fig. 3). Thus, the passage of the virtual point through these two anatomical planes defined three pulling phases (Fig. 4).

The obstetricians’ postures were then assessed at the passage of the first plane by calculating the height of the head as well as the joint angles of the hips, knees, ankles for the lower limbs, and of the trunk, shoulders, elbows and wrists for the upper limbs. To qualify the movement, the range of motion of these angles was computed during all three phases.

### Analysis

To define the different postures and techniques adopted by the obstetricians, the quantitative variables qualifying their posture and movements were processed by means of a principal component analysis using the RStudio software (RStudio Team. RStudio: Integrated Development Environment for R [Internet]. Boston, MA; 2020. Available from: http://www.rstudio.com/) in order to summarize the information obtained into a few principal dimensions. Then, an ascending hierarchical classification (AHC), consolidated using the K-means method, was applied to these results in order to objectify the existence of groups of similar behaviours. The categorical variable "experience" was processed using Chi-Squared tests to estimate the association of the level of experience with the groups of behaviours found.

### Ethical approval

Regarding its design, such a study does not require any ethical approval as stated by the Poitiers University Hospital Institutional Review Board. This study was declared to the Health Data Hub and the Data Protection Officer of the University Hospital of Poitiers (number F20210118141050). We collected free, informed consent from each volunteer before any investigation. The study was registered on https://clinicaltrials.gov (NCT04670380 –17/12/2020).

## Results

### Population characteristics

The obstetrician study group was composed of 38 women and 19 men, whose average age was 34 years (± 10 years), average height 170 cm (± 9 cm), and average weight 68 kg (± 14 kg). In terms of experience, 23 obstetricians were beginners (40%), 22 intermediates (39%), and 12 were experts (21%).

### Posture when crossing the first plane

The AHC made it possible to identify four clusters, thus corresponding to four groups of individuals sharing postural characteristics when crossing the first plane (Annex 1). Groups 1, 2, 3, and 4 were composed of 8 (14%), 12 (21%), 24 (42%) and 13 (23%) obstetricians, respectively. A table presenting the significance thresholds of the dominant variables is appended in Annex 2.

**Fig. 5:**
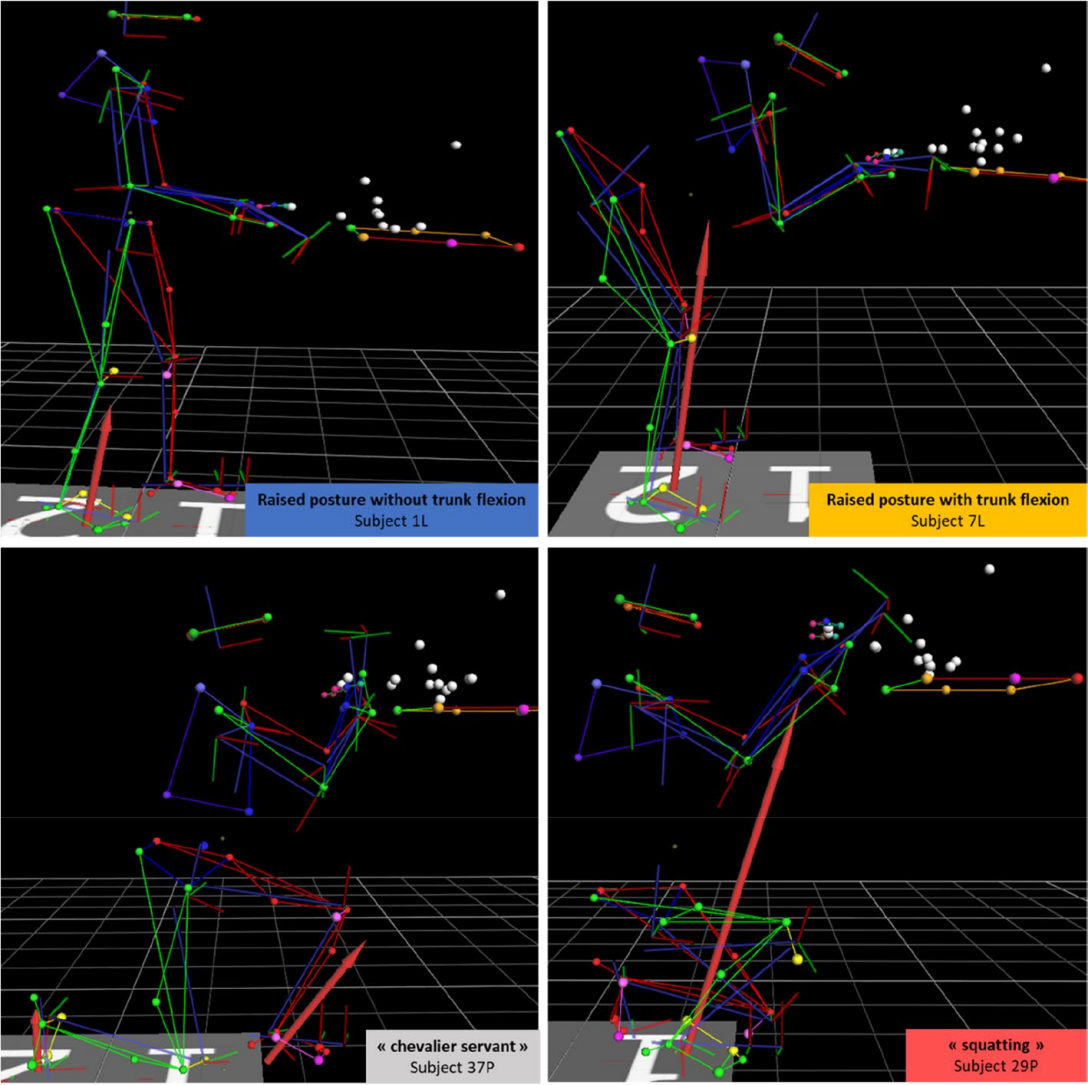
Representation of the most characteristic obstetrician of each group when crossing the first plane

One can note that the first and second groups of obstetricians assumed a raised posture (35% of the total population). The difference is that the second group of obstetricians flexed their chests strongly and therefore had their heads closer to the maternal perineum, while the heads of the first group of obstetricians were positioned well above the plane of the delivery table (Table 1; Fig. 5). Conversely, the third and fourth cluster obstetricians assumed a posture in which the lower limbs were strongly flexed (65% of the total population). For this group, the distance separating the head from the delivery table was small (Fig. 5).

**Table 1:**
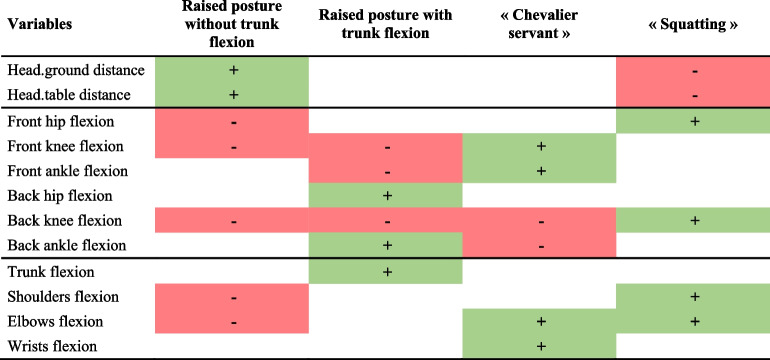
Summary table of differences between clusters when the first plane is crossed

Only significant variables in the sagittal plane with *p* < 0.01 are shown here. The values of the variables coloured in red are significantly smaller than for the rest of the subjects, while those coloured in green are larger. “Back” indicates that the joints belonged to the rear leg, “front”, to the front leg

### Movements during the first phase

The AHC made it possible to bring together three clusters in terms of movements performed during the first phase of foetal traction (Annex 3). Groups 1, 2, and 3 were composed of 31 (54%), 18 (32%), and 8 (14%) obstetricians, respectively. A table presenting the significance thresholds of the dominant variables is attached in the Appendix (Annex 4).

The first and second groups of obstetricians performed very few movements, conversely to the third group of obstetricians (Table 2). The first and second group of obstetricians initiated the foetal traction only after having settled into the posture of their choice, and maintained this position during the first plane. The difference between these two groups can be explained by greater wrist movements in the second group compared with the first. By contrast, the third group of obstetricians changed their posture (they flexed their lower limbs) while pulling.

**Table 2:**
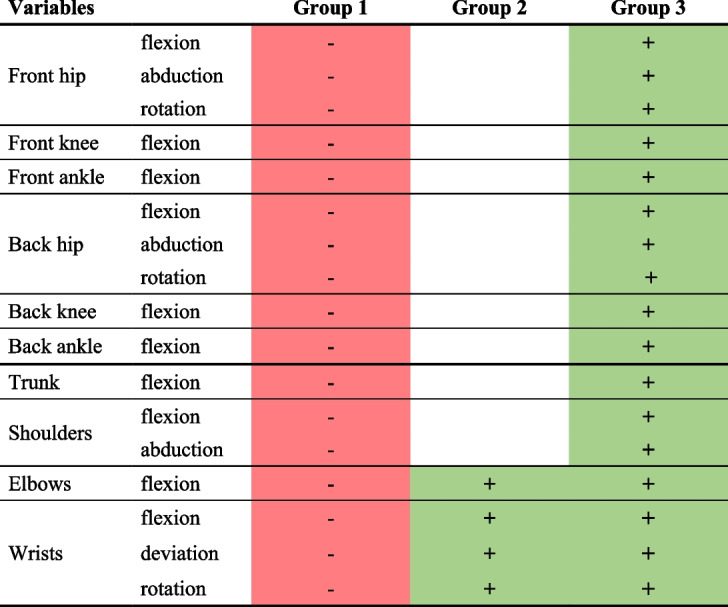
Summary table of differences between groups during the first phase

Only significant variables with *p* < 0.01 are shown here. The values of the variables coloured in red are significantly smaller than the rest of the subjects, while those coloured in green are larger. Back indicates the joints for the rear leg, and front for the front leg

### Movements during the second phase

According to the AHC, three clusters existed in terms of movements performed during the second phase (Annex 5). Groups 1, 2, and 3 were composed of 40 (70%), 11 (19%) and 6 (11%) obstetricians, respectively. A table presenting the significance thresholds for the dominant variables is attached in the Appendix (Annex 6).

According to Table 3, which summarizes the principal differences between the groups, the first group of obstetricians produced very few movements at their joints and therefore maintained a similar posture throughout this phase (70% of the total population). The second group of obstetricians generated a wider flexion of the upper limbs (19% of the total population). The third group of obstetricians generated a significant range of movement at all joints (11% of the total population).

**Table 3:**
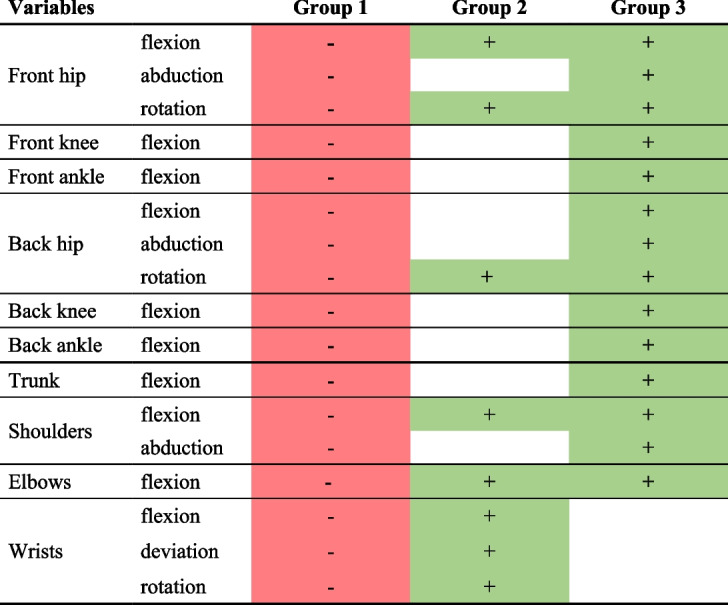
Summary table of differences between groups during the second phase

Only significant variables with *p* < 0.01 are shown here. The values of the variables coloured in red are significantly smaller than for the rest of the subjects, while those coloured in green are larger. Back indicates the joints for the rear leg and front for the front leg

### Movements from the third phase

The AHC made it possible to bring together three clusters in terms of movements performed during the third phase of foetal traction (Annex 7). Groups 1, 2, and 3 were composed of 23 (40%), 16 (28%), and 18 (32%) obstetricians, respectively. A table presenting the significance thresholds of the dominant variables is attached in the Appendix (Annex 8).

As can be deduced from Table 4, the first group of obstetricians produced very little movement at the joints except around the wrist and elbow joint: foetal head deflection was then caused by the action of the wrists (40% of the total population). The second group of obstetricians produced a large range of motion at the level of the knees: cephalic deflection was, in that case, generated by raising the lower limbs associated with the action of the shoulders and wrists (28% of the total population). The third group of obstetricians generated a greater range of motion than the second group of obstetricians because they performed a complete deflection of the foetal head (32% of the total population).

**Table 4:**
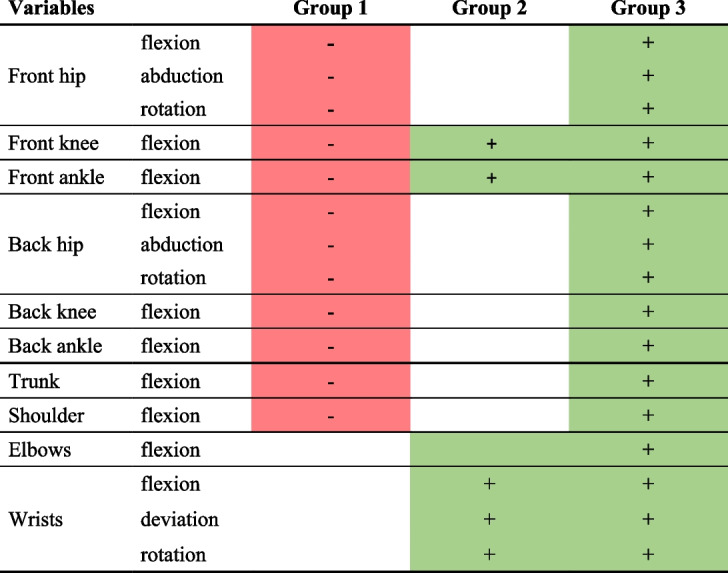
Summary table of differences between groups during the third phase

Only significant variables with *p* < 0.01 are shown here. The values of the variables coloured in red are significantly smaller than the rest of the subjects, while those coloured in green are larger. Back indicates the joints for the rear leg and front for the front leg

### Association between postures, movements, and level of experience

The number of experienced, intermediate, and beginner obstetricians was defined for each group (Table 5). A significant difference in their distribution was found according to the Chi-squared tests in the four groups describing posture (*p* < 0.05) as well as in the three groups corresponding to movements performed during the first phase of simulated childbirth (*p* < 0.05) as opposed to the groups corresponding to the last two phases of traction (*p* > 0.05).

**Table 5:** Level of experience found in each group revealed by the ACH

	**Group**	**Beginners**	**Intermediates**	**Experts**	**Total**
		***N* = 23**	***N* = 22**	***N* = 12**	***N* = 57**
**Posture** **(chi**^**2**^** = 0.027)**	1	22% (5)	14% (3)	0% (0)	14% (8)
	2	26% (6)	18% (4)	17% (2)	21% (12)
	3	48% (11)	45% (10)	25% (3)	42% (24)
	4	4% (1)	23% (5)	58% (7)	23% (13)
**Joint movement** **Phase 1** **(chi**^**2**^** = 0.047)**	1	39% (9)	54% (12)	84% (10)	54% (31)
	2	35% (8)	41% (9)	8% (1)	32% (18)
	3	26% (6)	5% (1)	8% (1)	14% (8)
**Joint movement** **Phase 2** **(chi**^**2**^** = 0.962)**	1	70% (16)	68% (15)	66% (8)	69% (39)
	2	17% (4)	23% (5)	17% (2)	19% (11)
	3	13% (3)	9% (2)	17% (2)	12% (7)
**Joint movement** **Phase 3** **(chi**^**2**^** = 0.688)**	1	39% (9)	41% (9)	42% (5)	40% (23)
	2	35% (8)	18% (4)	33% (4)	28% (16)
	3	26% (6)	41% (9)	25% (3)	32% (18)

Number of obstetricians according to the degree of experience belonging to the groups found to characterize the posture adopted when crossing the first plane, and to the groups found to describe the movements performed during the different phases

In terms of posture, 22% of the beginners, 14% of the intermediates but none of the experts were in the raised posture without trunk flexion, whereas the majority of experts were in the “squatting” position (58%) when crossing the first plan for only 4% of beginners and 23% of intermediates.

From the start of the traction until crossing the first plane, 84% of experts were in Group 1 corresponding to a low range of motion for only 39% of beginners and 54% of intermediates.

## Discussion

As mentioned in the introduction, the questions raised by this first study were threefold: to discover whether different techniques were adopted by the obstetricians, and, if yes, to define what distinguished these different techniques, and, finally, to establish whether the obstetricians’ experience is associated with the technique adopted. In the literature, little is indeed mentioned regarding the posture and movement the obstetricians should adopt to perform forceps delivery since only two papers contain references to them [12, 15]. This topic seems yet to be of interest for obstetricians, as attested by a discussion on the postures to adopt during forceps delivery on Researchgate [21].

The present study shows that four different postures were adopted when crossing the first plane and that very different pulling techniques were applied throughout delivery. Experts mainly positioned themselves in either a “chevalier servant” or a “squatting” posture before initiating traction, and made little movement until the foetal head was deflected. At the opposite end of the spectrum, many beginners initially stood, then changed posture at the beginning of delivery. It also appears that the amplitude of joint movements decreased with the level of expertise, which can partially be explained by the fact that very few expert subjects adopted a raised posture with or without trunk flexion and therefore did not need to modify their postures before traction.

The experts therefore mainly adopted either a very flexed “chevalier servant” posture or a “squatting” posture which enables observation at the level of the maternal perineum before initiating traction, as evidenced by the low height of the head relative to the delivery table obtained for these postures. By contrast, the majority of beginners adopted a raised posture, which does not allow good visibility of the pelvis or the perineum. To remedy this, the delivery table would have to be installed at a much higher level, which is not possible in practice.

Also, according to some recommendations [22], the traction should be performed following the umbilico-coxygeal axis, which is oriented downwards. From this point of view, the “standing without flexed torso” posture corresponding to group 1 does not seem compatible with a downward pull, the hands and forearms not being in this direction.

In addition, the experts had the tendency to make little movement to achieve the traction until the deflection of the foetal head, which also corresponds to the recommendations. Current practice advises removing the instrument when the small coronation is visualized that is to say when the foetal head appears at the vulva and takes the shape of a ring of approximately 5 cm in diameter. In fact, the installation of the forceps blades on the cephalic presentation increases the diameter of the latter and increases the risk of damage to the perineum during foetal release.

Finally, control of movements is favoured when the movements at the joints are small [23]. It therefore seems that the movements of the experts are to be preferred.

The abovementioned analysis elements of the different techniques adopted strongly suggest that, as has been shown for many interventions such as forceps blade placement, or the occurrence of obstetrical lesions of the anal sphincter during forceps deliveries [9, 24], the techniques for performing forceps delivery adopted by the experienced obstetricians in the present study seem better than those adopted by less experienced obstetricians.

A significant number of the young obstetricians in the present study, who were still in training, mentioned during the experiments a lack of training in instrumental deliveries during their internship. According to the literature, learning instrumental delivery using forceps on a mannequin makes it possible to both reduce obstetric lesions of the anal sphincter linked to deliveries performed before the obstetrician is sufficiently experienced [9], and to personalize the training of learners in the absence of any urgent and stressful situation [25]. Therefore, learning instrumental delivery using forceps on a mannequin could help to compensate for the drop in the number of forceps deliveries performed by obstetricians and its accompanying decline in skill acquisition. As emphasized in the literature, one has to learn to do better, and awareness of the importance of movements has positive effects on the performance of techniques [14, 26]. The development of simulation-based learning programs seems therefore essential for the transmission of knowledge, optimizing maternal-foetal safety, and would help to offset the decline in the number of forceps deliveries in current practice. Accordingly, the results from this present study provide some insight into those techniques that are more favourable and those that are less so.

Regarding the limitations of this study, the experimental conditions provided by the cameras and the markers were relatively imposing, and although the vast majority of obstetricians judged the realism of the manipulation to be satisfactory or excellent, one can naturally wonder whether this singular environment had an influence on the realism of the simulation. Also, the lubrication imposed by the manufacturer and necessary for the simulated delivery by forceps, was a source of slippage between the foetal head and the blades of the forceps. However, this should not affect the results presented above, since the trial that the obstetricians considered the most faithful to their clinical practice was selected for the analysis.

For the choice of statistical processing, the level at which the hierarchical tree was stopped can also be criticized. This threshold is justified by inertia and by the conjoint desire to explain as many differences as possible while maintaining clinical meaning in the groups created. Overall, we were able to obtain a fairly precise description of the different practices.

Concerning the recruitment, one could regret a lack of homogeneity in the numbers of obstetricians from Poitiers and Limoges (37 from Poitiers and 20 from Limoges) and in the numbers of obstetricians by level of experience (23 beginners, 22 intermediates, and 12 experts). The recruitment of a larger and better distributed set of obstetricians could make it possible to study with greater statistical power the influence of experience on the adopted practices. Another limit is that only two centres were included in this study. It is possible that very different practices could be identified in other centres. For instance, no obstetrician adopted a sitting posture, which was yet mentioned in the Researgate discussion [21] and in Leslie et al.’ work [14].

We also have to mention that the level of experience was based on the number of forceps deliveries that the physicians recalled. As a consequence, the accuracy of classification might be subject to “recall bias”. However, none of the subjects mentioned any doubt about this estimation. The other limit concerns the choice of this criterion itself since it is a quantitative and not a qualitative criterion taking into account the obstetrician’s results in terms of rate of fetal or maternal injury.

As some arguments have already been put forward to criticize some techniques adopted in the present study, such as the level of the head relative to the delivery table, it is now essential to qualify the practices observed by determining whether they ensure more or less safety for the mother and her child. To do this in the context of a similar experiment, the performance parameters for judging the different techniques observed could be the direction and intensity of the force produced by the obstetrician. Leslie et al.’ work therefore highlighted an association between posture and the traction force generated during the use of forceps in simulation [14]. Indeed, the study of the intensity of the pulling force produced by 55 obstetricians found that, on average, the participants pulled harder in a seated position than in a standing position. The measurement of the intensity of the force produced during foetal traction by forceps could then be different depending on the practices adopted. Furthermore, it has been established that excessive pulling forces are associated with the occurrence of obstetric lesions of the anal sphincter and neonatal lesions [27].

It will also be essential to characterise the posture and techniques adopted in terms of ergonomics by identifying the postures and techniques associated with lower musculoskeletal load on the locomotor system and by identifying those that favour the obstetrician’s balance.

Thus, the identification of the most favourable postures and movements would make it possible to develop training tools to optimize this technique and to ensure the maintenance of a number of experts in instrumental delivery by forceps. The mastery of the forceps by a sufficient number of obstetricians is the key to perpetuating this practice and limiting the occurrence of avoidable, serious maternal-foetal lesions.

However, it would in the future be important to renew this protocol with modifications, such as in the variety of presentation of the foetal head. Once the principles governing the optimized techniques will be identified, application in the delivery room would be possible to assess whether the risk of occurrence of perineal lesions does indeed decrease due to the use of the most favourable techniques during instrumental delivery by forceps.

## Conclusion

Obstetricians' movements during forceps delivery are potentially implicated in the occurrence of maternal perineal lesions. The results provided by this study reveal very different postures and movements, as well as a clear association between experience and practice. Many of the beginners were standing and had a large range of motion. Conversely, the experts mainly positioned themselves in either a “chevalier servant” or a “squatting” posture before initiating traction, and made little movement until the foetal head was deflected. The posture and movements adopted by the experts seemed more favourable for enabling observation at the level of the perineum, control of the movement, and a downward pull, as advised in obstetrics literature. This last hypothesis should, however, be verified by a new study of the pulling forces.

The present study has contributed to the identification of more favourable postures and movements to adopt when performing forceps delivery. The present results also emphasize the need for training tools, which seem fundamental to ensuring the maintenance of a number of experts in instrumental delivery by forceps. To perpetuate the mastery of this practice is essential to limit the occurrence of avoidable, serious maternal-foetal lesions.

### Supplementary Information

**Supplementary Material 1.**

**Supplementary Material 2.**

**Supplementary Material 3.**

**Supplementary Material 4.**

**Supplementary Material 5.**

**Supplementary Material 6.**

**Supplementary Material 7.**

**Supplementary Material 8.**

## Data Availability

The datasets used and/or analysed during the current study are available from the corresponding author on reasonable request.
