# Supplementary figures and images for: Analysis of the obstetrician's posture and movements during a simulated forceps delivery
Source: BMC Pregnancy Childbirth. 2024 Apr 8;24:253. doi: 10.1186/s12884-024-06457-4 (PMC11000395; doi:10.1186/s12884-024-06457-4)

Annex 1: Hierarchical classification of postures adopted when crossing the first plane


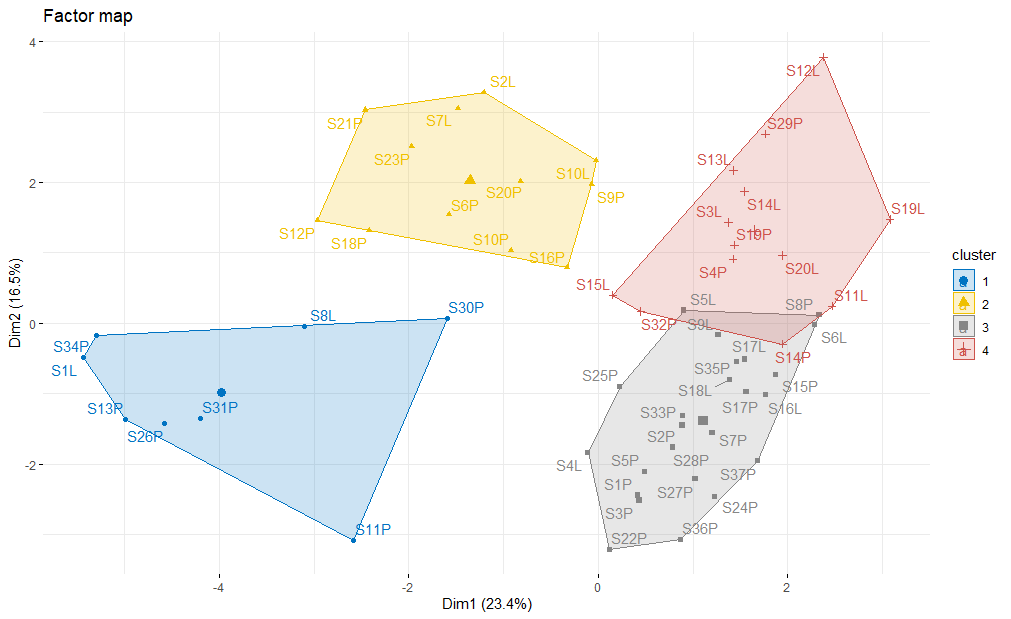

Supplement: Supplementary file 1 — Supplementary Material 1. [file 12884_2024_6457_MOESM1_ESM.docx]

*Annex 3: Hierarchical classification of gestures during the first phase*


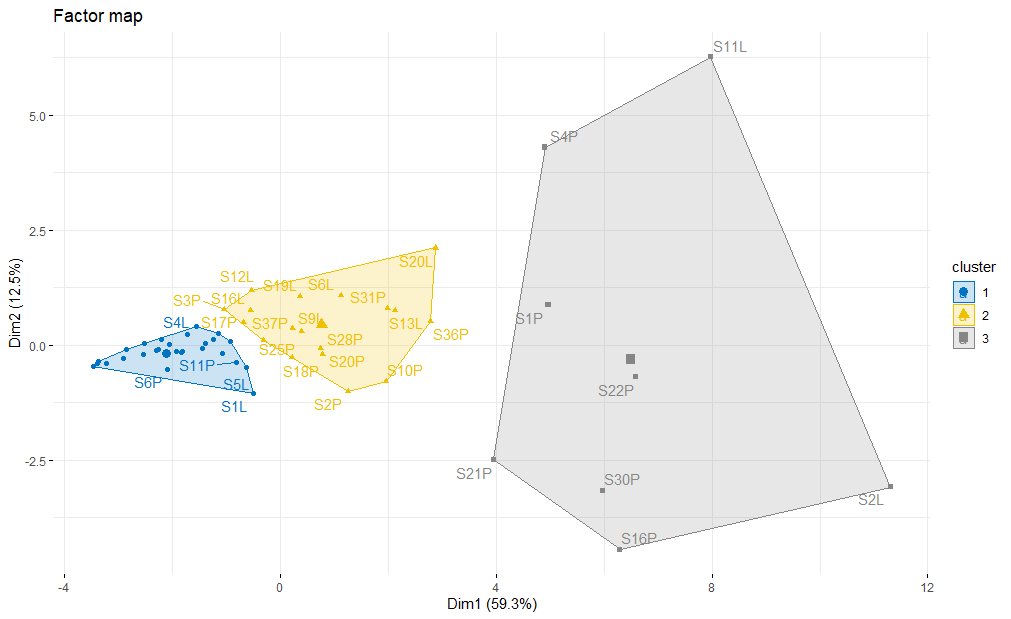

Supplement: Supplementary file 3 — Supplementary Material 3. [file 12884_2024_6457_MOESM3_ESM.docx]

Annex 5: Hierarchical classification of gestures during the second phase


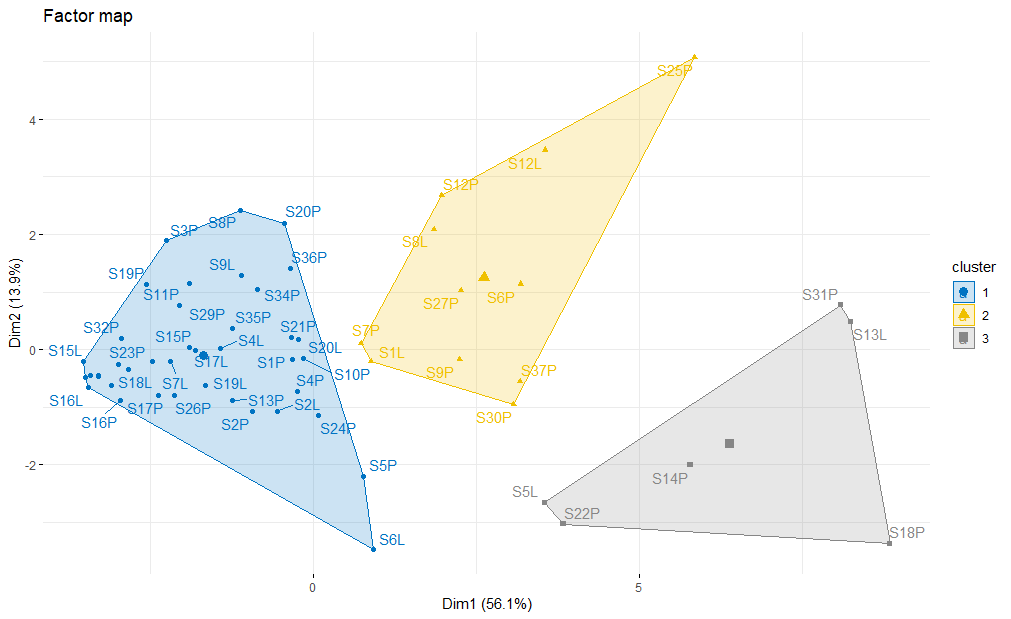

Supplement: Supplementary file 5 — Supplementary Material 5. [file 12884_2024_6457_MOESM5_ESM.docx]

Annex 7: Hierarchical classification of gestures during the third phase


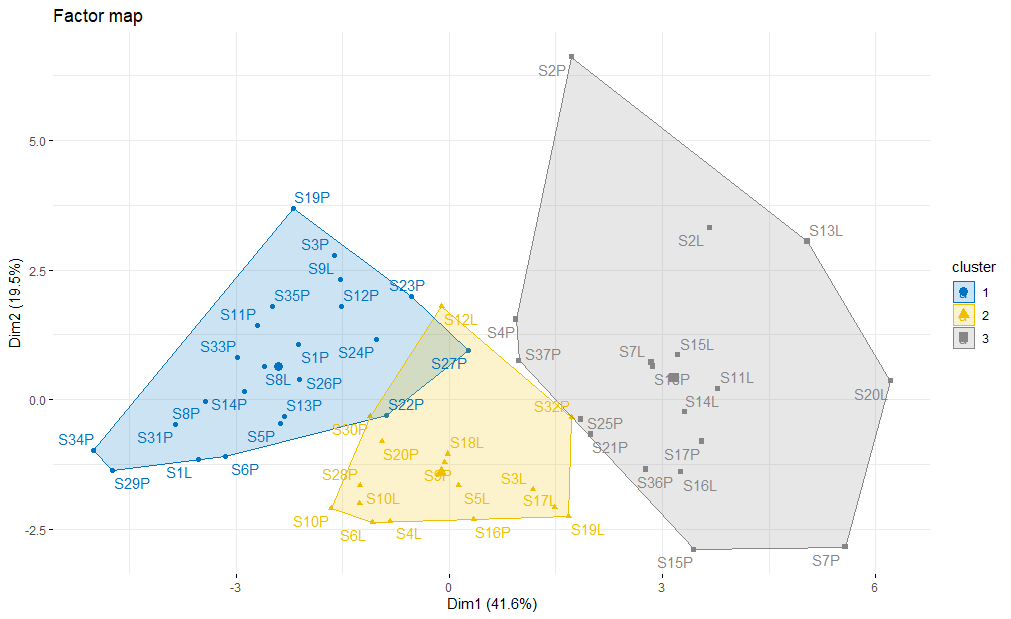

Supplement: Supplementary file 7 — Supplementary Material 7. [file 12884_2024_6457_MOESM7_ESM.docx]
