# Supplementary material for: Analysis of the obstetrician's posture and movements during a simulated forceps delivery
Source: BMC Pregnancy Childbirth. 2024 Apr 8;24:253. doi: 10.1186/s12884-024-06457-4 (PMC11000395; doi:10.1186/s12884-024-06457-4)
Supplement: Supplementary file 2 — Supplementary Material 2. [file 12884_2024_6457_MOESM2_ESM.docx]

*Annex 2:* *Variables justifying the creation of clusters when the first plane is crossed*

| Cluster | Variables | v.test | Mean in category | Overall mean | SD in category | Overall sd | p.value |
| --- | --- | --- | --- | --- | --- | --- | --- |
| 1 | Head.ground distance | 4,77 | 1,433 | 1,240 | 0,103 | 0,122 | 1,85E-06 |
|  | Head.table distance | 4,04 | 0,722 | 0,448 | 0,132 | 0,205 | 5,28E-05 |
|  | Wrists abduction | -2,50 | -1 | 17 | 19 | 22 | 1,24E-02 |
|  | Back hip flexion | -2,56 | 24 | 43 | 12 | 22 | 1,05E-02 |
|  | Shoulders flexion | -3,49 | 50 | 64 | 13 | 12 | 4,76E-04 |
|  | Elbows flexion | -3,61 | 58 | 84 | 25 | 22 | 3,01E-04 |
|  | Front knee flexion | -4,37 | 35 | 86 | 19 | 35 | 1,26E-05 |
|  | Back knee flexion | -4,61 | 42 | 102 | 19 | 40 | 3,96E-06 |
|  | Front hip flexion | -5,42 | 50 | 90 | 17 | 23 | 5,87E-08 |
| 2 | Thorax flexion | 5,11 | 51 | 31 | 14 | 15 | 3,24E-07 |
|  | Back hip flexion | 3,92 | 66 | 43 | 15 | 22 | 8,87E-05 |
|  | Back hip abduction | 3,12 | -2 | -10 | 8 | 9 | 1,82E-03 |
|  | Back ankle flexion | 2,75 | 32 | 19 | 11 | 18 | 5,88E-03 |
|  | Front knee flexion | -3,19 | 69 | 102 | 32 | 40 | 1,42E-03 |
|  | Back knee flexion | -3,50 | 54 | 86 | 24 | 35 | 4,64E-04 |
|  | Front ankle flexion | -3,62 | -5 | 9 | 9 | 15 | 2,96E-04 |
| 3 | Front hip abduction | 3,88 | 3 | -3 | 7 | 9 | 1,03E-04 |
|  | Front ankle flexion | 3,85 | 18 | 9 | 10 | 15 | 1,18E-04 |
|  | Front knee flexion | 3,35 | 104 | 86 | 12 | 35 | 8,21E-04 |
|  | Elbows flexion | 3,31 | 96 | 84 | 11 | 22 | 9,46E-04 |
|  | Wrists flexion | 2,86 | 34 | 26 | 11 | 19 | 4,21E-03 |
|  | Back knee flexion | 2,33 | 116 | 102 | 10 | 40 | 1,97E-02 |
|  | Back hip rotation | 2,16 | 2 | -2 | 9 | 12 | 3,05E-02 |
|  | Shoulders abduction | -2,00 | -3 | 9 | 23 | 37 | 4,50E-02 |
|  | Back hip abduction | -2,13 | -13 | -10 | 7 | 9 | 3,30E-02 |
|  | Front hip rotation | -2,44 | -8 | -2 | 11 | 14 | 1,48E-02 |
|  | Thorax flexion | -2,47 | 25 | 31 | 11 | 15 | 1,36E-02 |
|  | Back ankle flexion | -3,20 | 10 | 19 | 21 | 18 | 1,37E-03 |
|  | Back hip flexion | -3,62 | 30 | 43 | 14 | 22 | 3,00E-04 |
|  | Wrists rotation | -3,96 | -58 | -35 | 11 | 38 | 7,45E-05 |
| 4 | Back knee flexion | 4,18 | 143 | 102 | 16 | 40 | 2,98E-05 |
|  | Front hip flexion | 3,33 | 109 | 90 | 7 | 23 | 8,82E-04 |
|  | Shoulders flexion | 3,11 | 74 | 64 | 6 | 12 | 1,84E-03 |
|  | Front knee flexion | 3,08 | 113 | 86 | 23 | 35 | 2,07E-03 |
|  | Back hip flexion | 2,56 | 57 | 43 | 19 | 22 | 1,04E-02 |
|  | Wrists flexion | -2,02 | 16 | 26 | 18 | 19 | 4,38E-02 |
|  | Head.ground distance | -2,85 | 1 | 1 | 0 | 0 | 4,38E-03 |
|  | Head.table distance | -2,88 | 0 | 0 | 0 | 0 | 4,01E-03 |
|  | Front hip rotation | -3,12 | -9 | -3 | 10 | 9 | 1,81E-03 |
