# Supplementary material for: Analysis of the obstetrician's posture and movements during a simulated forceps delivery
Source: BMC Pregnancy Childbirth. 2024 Apr 8;24:253. doi: 10.1186/s12884-024-06457-4 (PMC11000395; doi:10.1186/s12884-024-06457-4)
Supplement: Supplementary file 4 — Supplementary Material 4. [file 12884_2024_6457_MOESM4_ESM.docx]

*Annex 4: Variables justifying the creation of clusters during the first phase*

| Cluster | Variable amplitude | v.test | Mean in category | Overall mean | SD in category | Overall sd | p.value |
| --- | --- | --- | --- | --- | --- | --- | --- |
| 1 | Back ankle flexion | -3,08 | 2 | 4 | 2 | 7 | 2,10E-03 |
|  | Shoulders abduction | -3,37 | 7 | 17 | 11 | 24 | 7,43E-04 |
|  | Front knee flexion | -3,47 | 3 | 9 | 4 | 14 | 5,20E-04 |
|  | Front hip abduction | -3,57 | 1 | 3 | 1 | 5 | 3,51E-04 |
|  | Front hip rotation | -3,68 | 1 | 4 | 1 | 5 | 2,36E-04 |
|  | Back hip abduction | -3,75 | 1 | 3 | 1 | 5 | 1,76E-04 |
|  | Back knee flexion | -3,91 | 4 | 12 | 5 | 18 | 9,24E-05 |
|  | Back hip rotation | -4,04 | 1 | 4 | 1 | 5 | 5,42E-05 |
|  | Front hip flexion | -4,22 | 2 | 7 | 2 | 10 | 2,46E-05 |
|  | Thorax flexion | -4,29 | 2 | 5 | 2 | 6 | 1,79E-05 |
|  | Front ankle flexion | -4,41 | 2 | 5 | 2 | 5 | 1,05E-05 |
|  | Elbows flexion | -4,45 | 2 | 5 | 2 | 5 | 8,77E-06 |
|  | Back hip flexion | -4,58 | 3 | 8 | 3 | 10 | 4,62E-06 |
|  | Shoulders flexion | -4,82 | 2 | 6 | 2 | 6 | 1,43E-06 |
|  | Wrists flexion | -5,29 | 3 | 8 | 3 | 7 | 1,21E-07 |
|  | Wrists rotation | -5,38 | 3 | 8 | 2 | 7 | 7,49E-08 |
|  | Wrists abduction | -5,57 | 2 | 4 | 2 | 3 | 2,48E-08 |
| 2 | Wrists abduction | 4,02 | 7 | 4 | 2 | 3 | 5,86E-05 |
|  | Wrists flexion | 3,16 | 12 | 8 | 6 | 7 | 1,56E-03 |
|  | Wrists rotation | 2,78 | 12 | 8 | 5 | 7 | 5,41E-03 |
|  | Elbows flexion | 2,10 | 7 | 5 | 5 | 5 | 3,59E-02 |
| 3 | Front hip flexion | 5,80 | 27 | 7 | 14 | 10 | 6,53E-09 |
|  | Front knee flexion | 5,76 | 35 | 9 | 20 | 14 | 8,36E-09 |
|  | Back knee flexion | 5,64 | 45 | 12 | 24 | 18 | 1,67E-08 |
|  | Back ankle flexion | 5,54 | 16 | 4 | 10 | 7 | 3,01E-08 |
|  | Back hip flexion | 5,53 | 26 | 8 | 13 | 10 | 3,12E-08 |
|  | Back hip rotation | 5,31 | 13 | 4 | 8 | 5 | 1,11E-07 |
|  | Back hip abduction | 5,24 | 12 | 3 | 8 | 5 | 1,56E-07 |
|  | Thorax flexion | 5,24 | 15 | 5 | 8 | 6 | 1,64E-07 |
|  | Front hip rotation | 4,97 | 11 | 4 | 8 | 5 | 6,74E-07 |
|  | Front hip abduction | 4,81 | 10 | 3 | 8 | 5 | 1,54E-06 |
|  | Shoulders flexion | 4,63 | 15 | 6 | 8 | 6 | 3,58E-06 |
|  | Front ankle flexion | 4,52 | 12 | 5 | 7 | 5 | 6,19E-06 |
|  | Wrists rotation | 3,99 | 17 | 8 | 7 | 7 | 6,59E-05 |
|  | Shoulders abduction | 3,77 | 47 | 17 | 40 | 24 | 1,65E-04 |
|  | Elbows flexion | 3,57 | 11 | 5 | 5 | 5 | 3,62E-04 |
|  | Wrists flexion | 3,35 | 15 | 8 | 6 | 7 | 7,96E-04 |
|  | Wrists abduction | 2,62 | 7 | 4 | 3 | 3 | 8,91E-03 |
