# Supplementary material for: Analysis of the obstetrician's posture and movements during a simulated forceps delivery
Source: BMC Pregnancy Childbirth. 2024 Apr 8;24:253. doi: 10.1186/s12884-024-06457-4 (PMC11000395; doi:10.1186/s12884-024-06457-4)
Supplement: Supplementary file 6 — Supplementary Material 6. [file 12884_2024_6457_MOESM6_ESM.docx]

Annex 6: Variables justifying the creation of clusters during the second phase

| Cluster | Variable amplitude | v.test | Mean in category | Overall mean | SD in category | Overall sd | p.value |
| --- | --- | --- | --- | --- | --- | --- | --- |
| 1 | Shoulders abduction | -2,90 | 21 | 31 | 23 | 36 | 3,70E-03 |
|  | Back ankle flexion | -3,58 | 4 | 7 | 5 | 8 | 3,43E-04 |
|  | Wrists abduction | -3,78 | 7 | 9 | 4 | 7 | 1,57E-04 |
|  | Back knee flexion | -3,79 | 15 | 23 | 21 | 25 | 1,53E-04 |
|  | Back hip flexion | -3,81 | 9 | 13 | 9 | 11 | 1,37E-04 |
|  | Front hip abduction | -4,32 | 3 | 4 | 2 | 4 | 1,58E-05 |
|  | Wrists flexion | -4,54 | 15 | 23 | 13 | 20 | 5,63E-06 |
|  | Wrists rotation | -4,63 | 14 | 22 | 11 | 19 | 3,59E-06 |
|  | Front ankle flexion | -4,65 | 6 | 9 | 4 | 9 | 3,24E-06 |
|  | Thorax flexion | -4,81 | 6 | 9 | 4 | 8 | 1,48E-06 |
|  | Back hip abduction | -4,86 | 3 | 5 | 3 | 5 | 1,15E-06 |
|  | Elbows flexion | -4,87 | 8 | 11 | 5 | 8 | 1,14E-06 |
|  | Front knee flexion | -5,07 | 8 | 18 | 9 | 22 | 3,94E-07 |
|  | Back hip rotation | -5,11 | 4 | 6 | 2 | 6 | 3,22E-07 |
|  | Front hip rotation | -5,76 | 4 | 6 | 3 | 5 | 8,53E-09 |
|  | Front hip flexion | -5,99 | 7 | 13 | 5 | 11 | 2,07E-09 |
|  | Shoulders flexion | -6,18 | 8 | 12 | 4 | 8 | 6,45E-10 |
| 2 | Wrists flexion | 4,20 | 46 | 23 | 20 | 20 | 2,64E-05 |
|  | Wrists abduction | 4,18 | 17 | 9 | 9 | 7 | 2,93E-05 |
|  | Shoulders flexion | 4,12 | 20 | 12 | 5 | 8 | 3,76E-05 |
|  | Wrists rotation | 3,87 | 42 | 22 | 21 | 19 | 1,10E-04 |
|  | Front hip rotation | 3,26 | 10 | 6 | 3 | 5 | 1,13E-03 |
|  | Elbows flexion | 3,19 | 18 | 11 | 6 | 8 | 1,44E-03 |
|  | Front hip flexion | 2,81 | 21 | 13 | 5 | 11 | 5,02E-03 |
|  | Back hip rotation | 2,57 | 11 | 6 | 4 | 6 | 1,01E-02 |
| 3 | Front knee flexion | 5,96 | 69 | 18 | 18 | 22 | 2,50E-09 |
|  | Front ankle flexion | 5,67 | 29 | 9 | 9 | 9 | 1,45E-08 |
|  | Front hip flexion | 5,32 | 36 | 13 | 7 | 11 | 1,01E-07 |
|  | Back hip abduction | 5,09 | 15 | 5 | 5 | 5 | 3,57E-07 |
|  | Front hip abduction | 5,02 | 13 | 4 | 7 | 4 | 5,28E-07 |
|  | Thorax flexion | 4,72 | 24 | 9 | 10 | 8 | 2,35E-06 |
|  | Front hip rotation | 4,40 | 14 | 6 | 3 | 5 | 1,10E-05 |
|  | Back knee flexion | 4,37 | 64 | 23 | 11 | 25 | 1,26E-05 |
|  | Back hip rotation | 4,31 | 16 | 6 | 9 | 6 | 1,65E-05 |
|  | Shoulders flexion | 3,91 | 23 | 12 | 3 | 8 | 9,20E-05 |
|  | Back ankle flexion | 3,69 | 18 | 7 | 11 | 8 | 2,25E-04 |
|  | Back hip flexion | 3,59 | 29 | 13 | 13 | 11 | 3,29E-04 |
|  | Elbows flexion | 3,15 | 21 | 11 | 10 | 8 | 1,61E-03 |
|  | Shoulders abduction | 2,66 | 68 | 31 | 71 | 36 | 7,92E-03 |
