# Supplementary material for: Analysis of the obstetrician's posture and movements during a simulated forceps delivery
Source: BMC Pregnancy Childbirth. 2024 Apr 8;24:253. doi: 10.1186/s12884-024-06457-4 (PMC11000395; doi:10.1186/s12884-024-06457-4)
Supplement: Supplementary file 8 — Supplementary Material 8. [file 12884_2024_6457_MOESM8_ESM.docx]

Annex 8: Variables justifying the creation of clusters during the third phase

| Cluster | Variable amplitude | v.test | Mean in category | Overall mean | SD in category | Overall sd | p.value |
| --- | --- | --- | --- | --- | --- | --- | --- |
| 1 | Back ankle flexion | -2,55 | 13 | 20 | 14 | 19 | 1,09E-02 |
|  | Shoulders flexion | -3,02 | 19 | 27 | 8 | 17 | 2,56E-03 |
|  | Thorax flexion | -3,12 | 11 | 16 | 7 | 9 | 1,81E-03 |
|  | Front hip rotation | -3,94 | 7 | 12 | 4 | 8 | 8,22E-05 |
|  | Back hip rotation | -4,20 | 5 | 12 | 3 | 10 | 2,72E-05 |
|  | Front hip abduction | -4,25 | 4 | 9 | 2 | 7 | 2,15E-05 |
|  | Back hip abduction | -4,46 | 5 | 10 | 3 | 8 | 8,08E-06 |
|  | Back hip flexion | -4,51 | 17 | 31 | 10 | 20 | 6,44E-06 |
|  | Front ankle flexion | -5,14 | 8 | 16 | 5 | 8 | 2,81E-07 |
|  | Back knee flexion | -5,38 | 26 | 60 | 18 | 39 | 7,26E-08 |
|  | Front knee flexion | -5,54 | 17 | 45 | 13 | 32 | 3,05E-08 |
|  | Front hip flexion | -5,76 | 16 | 38 | 11 | 24 | 8,42E-09 |
| 2 | Front knee flexion | 2,81 | 65 | 45 | 23 | 32 | 4,92E-03 |
|  | Front ankle flexion | 2,42 | 20 | 16 | 6 | 8 | 1,54E-02 |
|  | Wrists abduction | 2,81 | 13 | 27 | 9 | 22 | 4,93E-03 |
|  | Wrists rotation | 3,02 | 27 | 52 | 17 | 40 | 2,55E-03 |
|  | Wrists flexion | 3,73 | 25 | 48 | 16 | 29 | 1,88E-04 |
| 3 | Back hip rotation | 5,92 | 24 | 12 | 9 | 10 | 3,31E-09 |
|  | Back hip abduction | 5,68 | 18 | 10 | 6 | 8 | 1,33E-08 |
|  | Front hip abduction | 5,51 | 16 | 9 | 7 | 7 | 3,57E-08 |
|  | Front hip rotation | 4,88 | 19 | 12 | 8 | 8 | 1,06E-06 |
|  | Thorax flexion | 4,73 | 25 | 16 | 9 | 9 | 2,27E-06 |
|  | Front hip flexion | 4,32 | 59 | 38 | 19 | 24 | 1,57E-05 |
|  | Shoulders flexion | 3,93 | 40 | 27 | 22 | 17 | 8,53E-05 |
|  | Back knee flexion | 3,82 | 89 | 60 | 32 | 39 | 1,31E-04 |
|  | Back hip flexion | 3,82 | 46 | 31 | 18 | 20 | 1,34E-04 |
|  | Back ankle flexion | 3,77 | 34 | 20 | 20 | 19 | 1,65E-04 |
|  | Front knee flexion | 3,13 | 65 | 45 | 28 | 32 | 1,77E-03 |
|  | Front ankle flexion | 3,08 | 21 | 16 | 7 | 8 | 2,08E-03 |
|  | Elbows flexion | 2,95 | 31 | 22 | 17 | 14 | 3,15E-03 |
|  | Wrists abduction | 2,53 | 38 | 27 | 31 | 22 | 1,14E-02 |
|  | Wrists rotation | 2,47 | 72 | 52 | 50 | 40 | 1,35E-02 |
|  | Wrists flexion | 2,04 | 59 | 48 | 24 | 29 | 4,16E-02 |
